# Effectiveness of Inactivated COVID-19 Vaccination Against COVID-19–Related Hospitalization and Severe Outcomes in Adults ≥80 Years During Omicron Circulation in Beijing, China: Retrospective Cohort Study

**DOI:** 10.2196/82915

**Published:** 2026-03-11

**Authors:** Dan Zhao, Ying Ma, Juan Li, Xiaomei Li, Zhiqiang Cao, Wei Yao, Jiang Wu, Luodan Suo

**Affiliations:** 1 Beijing Key Laboratory of Surveillance, Early Warning and Pathogen Research on Emerging Infectious Diseases, Beijing Center for Disease Prevention and Control, 16 Hepingli Middle St, Dongcheng District, Beijing, 100013, China, 86 01064407095; 2 Beijing Research Center for Respiratory Infectious Diseases, Beijing, China; 3 School of Public Health, Capital Medical University, Beijing, China

**Keywords:** older individuals, COVID-19, vaccine effectiveness, hospitalization, booster dose, real-world evidence

## Abstract

**Background:**

A large wave of COVID-19 caused by SARS-CoV-2 Omicron subvariants began in Beijing in early December 2022.

**Objective:**

This study aimed to evaluate the COVID-19 vaccine effectiveness (VE) in mitigating the risk of COVID-19–related hospitalization during the epidemic.

**Methods:**

We conducted a retrospective cohort study linking regional health care data and vaccination registry routinely collected in Beijing. All electronic medical records on COVID-19–related hospital discharges of older inpatients aged ≥80 years during November 2022 and February 2023 were included. Poisson regressions were used to estimate incidence risk ratio of COVID-19–related hospitalization, severe or critical cases, and in-hospital death compared with unvaccinated groups, adjusting for gender and age. VE was calculated as 1 minus incidence risk ratio×100%.

**Results:**

A total of 53,789 individuals aged ≥80 years were included, 28,423 (52.84%) were male, 45,270 (84.16%) were aged 80‐89 years, and 8519 (15.84%) were aged ≥90 years. Overall, 30,531 (56.76%) were in the vaccine group, with 4524 (8.41%) of the total participants receiving partial vaccination, 20.91% completing the primary series, and 14,761 (27.44%) receiving one booster. Additionally, 23,258 (43.24%) were in the unvaccinated group. Of the 53,789 hospitalized individuals, 17,916 (33.31%) had a COVID-19 diagnosis, 3535 (6.57%) had COVID-19–related hospitalization, 961 (1.79%) were COVID-19 severe or critical cases, and 4130 (7.68%) had in-hospital death. The analysis revealed that the VE of booster vaccination in preventing COVID-19–related hospital, severe or critical COVID-19, and in-hospital death was 63.5% (95% CI 59.8%‐66.9%), 66.9% (95% CI 60.1%‐72.6%) and 79.4% (95% CI 77%‐81.5%), the VE of primary series 56% (95% CI 51.4%‐60.2%), 66.8% (95% CI 59%‐73%) and 66.4% (95% CI 63%‐69.5%).

**Conclusions:**

The first booster vaccination was associated with significantly reduced the risk of COVID-19–related severe outcomes in older inpatients aged ≥80 years during the Omicron-dominant period. Considering the potential selection bias and unmeasured confounders, these estimates may reflect both the VE and the better baseline health status of the vaccinated older individuals.

## Introduction

COVID-19 vaccines have saved millions of lives worldwide by providing strong protection against serious illness, hospitalization, and death [1]. As SARS-CoV-2 evolves, changes in the receptor-binding domain of the spike protein are capable of evading antibodies induced by vaccination or infection; this waning immunity and reduced vaccine effectiveness (VE) have been observed [2,3]. Since 2022, SARS-CoV-2 Omicron and its sub-lineages (including BA.1, BA.2, BA.4, BA.5, XBB, BQ.1, and JN.1) have emerged and become the dominant circulating variants worldwide [4]. A serological study demonstrated that 3 to 5 doses of the ancestral monovalent vaccine elicited neutralizing antibodies with a narrow breadth against Omicron BA.5 and BA.2.75, while failing to neutralize BQ.1.1 and XBB sublineages [5]. Although severe COVID-19 and death are currently less prevalent in the Omicron-dominant era than in previous years [5], SARS-CoV-2 continues to cause significant morbidity and mortality, especially among the older population and those with existing medical conditions, including high blood pressure, diabetes, obesity, immunosuppression, cancer, and pregnancy [1]. The World Health Organization recommends that older adults as a priority group should be included in the long-term vaccine immunization strategy. However, as the continuous evolution of SARS-CoV-2 persists, it is probable that both the currently available and newly updated variant-specific vaccines will fall behind the viral antigenic evolution [6,7].

In China, the COVID-19 vaccine was approved for emergency use in July 2020, and a territory-wide massive vaccination program was launched, first in all adults aged 18 years and older at the end of March 2021, then expanding to adolescents aged 12‐17 years in July 2021, and to children 3‐11 years in October 2021. The coverage of the primary COVID-19 vaccine among the entire population, individuals aged ≥60 years, and those aged ≥80 years was estimated at 90.4%, 86.6%, and 66.4%, respectively [8]. Randomized clinical trials have shown that COVID-19 vaccines provided moderate protection against SARS-CoV-2 infection [9-14]. Post-market studies have shown moderate or high levels of protection against severe illness, hospitalization, and death in Chinese adults [15,16]. However, few data are available for those aged 80 years and older, especially regarding the risk of COVID-19–related hospitalization after the first booster dose during the Omicron period. Older people are at a higher risk of severe disease outcomes from COVID-19 infection, with higher rates of hospitalization and mortality than other ages, even after receiving the booster dose [17,18]. To guide the rational use of COVID-19 vaccines, it is crucial to evaluate and update their real-world effectiveness of COVID-19 vaccines, especially in older adults.

Beijing is the capital city of China, with a population of 21.8 million in 2024. According to the Beijing Immunization Information Management System data, inactivated vaccines constituted 99.55% (63.52 million doses) of Beijing’s administered COVID-19 vaccination through November 2022. China, including Beijing, adhered to a “zero-COVID-19” policy from April 2020 to early December 2022, which interrupted the transmission of SARS-CoV-2 in varying-scale COVID-19 outbreaks caused by variants [19]. Since December 2022, the zero-COVID policy has been rapidly lifted nationwide, with a major wave of Omicron sublineages. In Beijing, the cumulative infection attack rate was estimated to be 75.7% [20,21], caused by the Omicron BF.7 sub-lineage primary [22]. The roll-out of the fourth vaccine dose commenced in December 2022. In this study, we assessed the real-world VE of COVID-19 vaccines against COVID-19–related hospitalization during the Omicron wave in a well-traced cohort of older adults aged ≥80 years in Beijing.

## Methods

### Study Population and Design

We conducted a retrospective cohort study in Beijing. This study included Beijing residents enrolled in the Beijing Hospital Utilization Information System (BHUIS), an electronic platform managed by the Beijing Municipal Health Commission’s Information Center (BMHCIC). The system aggregates standardized inpatient demographic and discharge records from all tertiary and secondary hospitals, as well as more than 50% of primary care facilities across the city, capturing 94% of all hospital admissions in Beijing between 2022‐2023. In this study, individuals recorded in BHUIS were eligible for inclusion if he or she was aged ≥80 years (with a birth date on or before November 1, 1942), residents of Beijing, and admitted to the hospital between November 1, 2022 and February 28, 2023 (the starting and ending date of the Omicron wave). We excluded individuals who met any one criteria: individuals who had a previous COVID-19 infection before November 1, 2022 recorded in the National Notifiable Infectious Disease Surveillance System (NNIDSS); those who had any COVID-19 vaccination recorded after November 1, 2022, to avoid misclassification of exposure status due to overlapping seroconversion periods and outcome observation windows; those who received four doses of COVID-19 vaccine, as this subgroup (n=424) was undersampled for robust statistical analysis and risked exposure misclassification given their distinct vaccination timeline (eg, fourth doses administered since December in 2022).

### Data Source

We obtained complete electronic medical records of older adults aged ≥80 years who were admitted to the hospital between November 1, 2022 and February 28, 2023 from the BMHCIC. BMHCIC performed data queries and abstraction in BHUIS on December 30, 2023, to account for delays in reporting. All hospital discharges in the study cohort were downloaded and included in the study. The retrieved variables included patient name, age, identification card number, hospital case number, in-hospital deaths, discharge diagnosis (principal and secondary diagnosis), date of admission, and postal code of the residing address. To avoid duplicated hospitalizations, we retained records with the earliest admission date of the same individual who had multiple hospitalizations during a 21-day period. For records occurring 21 days apart, we considered it a new event of hospitalization for the same person.

We collected the vaccination history of individuals (ie, vaccine type, dosage, and date of injection) from the Beijing Center for Disease Control and Prevention, by linking hospitalization records with the Beijing Immunization Information Management System (IIS). The IIS is a web-based system akin to other computerized population-based databases previously documented in the literature [23], in which COVID-19 vaccine recipient information can be readily queried and retrieved using the vaccinee’s identification card number as an identifier. A “double match” technique, using an individual’s name and identification card number as identifiers, was used to link and acquire individual’s COVID-19 vaccine exposure. In our study, vaccine information recorded in the IIS can be viewed as a complete record of the study cohort, as every dose of COVID-19 vaccine administered in the administrative region was required to be entered into the IIS by nurses through client application software.

### Exposures

Based on the individual’s COVID-19 vaccine history before the Omicron wave started, we categorized the study cohort into four groups, (1) unvaccinated (no history of COVID-19 vaccination before November 1, 2022), (2) partially vaccinated (≥14 d after receipt of the first vaccine dose and before completing the full primary series), (3) completed primary series (≥14 d after completing the full primary series and before receipt of the first booster dose), and (4) completed the first booster vaccination (≥7 d after receipt of the booster dose). According to China’s COVID-19 Vaccines Immunization Schedule, the primary series of COVID-19 vaccine entailed two doses of inactivated vaccine, one dose of adenovirus vector vaccine, or three doses of recombination vaccine, while the booster vaccination involved an additional one dose of inactivated vaccine, adenovirus vector vaccine, or recombination vaccine after the recipient had completed the primary series.

### Outcomes and Covariates

The primary study outcome was hospitalization with COVID-19–related disease, defined as hospital discharge diagnosed with code U07.1 in the *International Statistical Classification of Diseases and Related Health Problems 10th Revision, 10th Revision (ICD-10)*. All hospitalizations with COVID-19 had a positive nucleic acid amplification test. Other outcomes evaluated in the study included COVID-19–related hospitalization, severe or critical illness, and in-hospital death. Considering the high infection rate of SARS-CoV-2, only inpatients with COVID-19 *ICD-10* code in “principal diagnosis” were defined as COVID-19–related hospitalization. Severe or critical illness was assessed by doctors based on the criteria provided in the Diagnosis and Treatment Protocol for COVID-19 (Version 10) [24]. Severe illness was defined as any of the following: (1) respiratory distress (ie, respiration rate ≥30 breaths per min), (2) oxygen saturation ≤93 % at rest, (3) the ratio of arterial partial pressure of oxygen and fraction of inspired oxygen ≤300 mmHg, or (4) chest imaging showing obvious lesion progression (>50% increase within 24‐48 h). Critical COVID-19 was defined as any of the following: (1) respiratory failure requiring mechanical ventilation, (2) shock, or (3) other organ failure requiring intensive care unit admission. In-hospital death with COVID-19 was defined as the death record of inpatients with COVID-19–related disease, the use of all-cause in-hospital mortality in patients diagnosed with COVID-19 cannot represent or estimate the specific COVID-19 mortality, due to the inability to definitively attribute death causation using available health care data. We controlled for several patient-level characteristics (ie, sex and age) by stratifying the analysis of the association between vaccination and outcome within these variables.

### Statistics Analysis

For comparison of baseline characteristics, we used the chi-square test or Fisher exact test for categorical variables as appropriate and Bonferroni method for their pairwise comparisons, used Kruskal-Wallis test for continuous variables and Mann-Whitney *U* test for their pairwise comparisons. We calculated the risk of COVID-19 among hospitalized patients during the study period by dividing the total number of inpatients diagnosed with COVID-19–related diseases by the size of the study cohort. The 95% CI was estimated using the normal approximation of Poisson distribution. The association (ie, incidence risk ratio) between vaccination and COVID-19–related hospitalization, severe or critical illness, and death was evaluated by comparing the risk between vaccinated and unvaccinated groups using Poisson regression models with a robust variance estimator, adjusting for sex and age. The VE was estimated as ([1-incidence risk ratio]×100%) for partially vaccinated, primary series, and booster vaccination. We conducted a sensitivity analysis restricting the outcome to hospitalizations with severe or critical COVID-19 as the principal diagnosis. Data cleaning was performed in MySQL 5.7 because of its high-performance data management capabilities. All statistical analyses were conducted using SPSS 20.0 (IBM Corp). All *P* values were 2-sided and considered significant at *P*<.05. Considering that our data consisted of almost all inpatient cases during the specified time, and 33,724 excluded records reflected cleaning and cohort eligibility criteria (Figure S1) in Multimedia Appendix 1, no survey weights were used in the analyses.

### Ethical Considerations

The study was conducted in accordance with the Declaration of Helsinki and approved by the Human Research Ethics Committee of the Beijing Center for Disease Prevention and Control (approval number: 2022‐14), the retrospective analysis without additional informed consent was approved by the review board. To safeguard participant information, all the data were deidentified after linkage. Additionally, access to the data was restricted to authorized research personnel only.

## Results

### Basic Characteristics of Study Population

**Figure 1.**
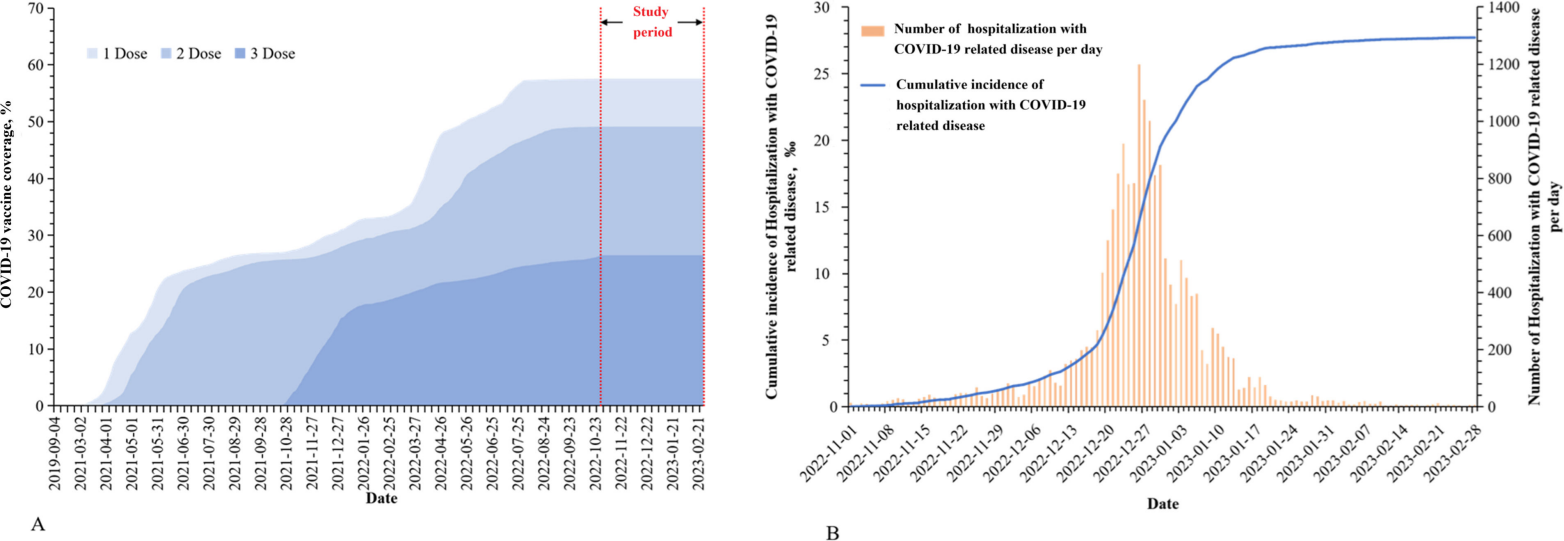
Temporal trends in COVID-19 vaccination coverage and hospitalization with COVID-19 incidence among adults aged ≥80 years from November 2022 to February 2023 in Beijing, China. (A) Cumulative daily coverage of COVID-19 vaccination, with the x-axis representing the vaccination date. This data aims to show the progression of vaccination over time. (B) The number and cumulative incidence of hospitalizations with COVID-19, where the x-axis indicates the admission date.

A total of 87,513 records were registered in the study. After removing 28,551 duplicate case records (84.7% of exclusions), 1570 (4.6%) whose birthday did not meet the criteria, and 3603 (10.7%) who were vaccinated between November in 2022 and February in 2023 and ineligible for inclusion, 53,789 individuals were included in the study cohort. Of these, 28,423 (52.84%) were male. A total of 30,531 (56.76%) belonged to the vaccination group, while 23,258 (43.24%) were unvaccinated, as detailed in Table 1. The vaccine rollout in the study cohort was shown in Figure 1.

**Table 1.** Basic characteristics of the hospitalized older patients with COVID-19 cohort by vaccination status in Beijing during the Omicron wave from November 2022 to February 2023.

Characteristics	Total, n (%)	Unvaccinated, n (%)	Partially vaccinated, n (%)	Primary vaccination, n (%)	Booster vaccination, n (%)	Chi-square (*df*)/ *H* value	*P* value
No. of persons	53,789	23,258 (43.24)	4524 (8.41)	11,246 (20.91)	14,761 (27.44)	—^e^	—^e^
Sex							
Male	28,423 (52.84)	11,723 (50.40)^a^^(1)^	2177 (48.12)^a^^(2)^	5647 (50.21)^a^^(1,2)^	8876 (60.13)^a^^(3)^	44.86^b^^,^^c^	<.001
Female	25,366 (47.16)	11,535 (49.60)^a^^(1)^	(51.88)^a^^(2)^	99 (49.79)^a^^(1,2)^	5885 (39.87)^a^^(3)^		
Age (years), median (IQR)	85 (82-88)	86 (83-89)^a^^(1)^	85 (82-88)^a^^(2)^	85 (82-88)^a^^(2)^	83 (81-86)^a^^(3)^	2515.06^d^	<.001
Age group (years)							
80-89	45,270 (84.16)	18,126 (77.93)^a^^(1)^	3819 (84.42)^a^^(2)^	9621 (85.55)^a^^(3)^	13,704 (92.84)^a^^(4)^	1526.99^b^^,^^c^	<.001
90+	8519 (15.84)	5132 (22.07)^a^^(1)^	705 (15.58)^a^^(2)^	1625 (14.45)^a^^(3)^	1057 (7.16)^a^^(4)^		

a indicated the result of pairwise comparisons, if two vaccination groups have the same superscript number, that means there is no significant difference between them; if they are different, there is a significant difference (*P*<.05).

b Chi-square value.

c Degree of freedom (*df*)=3.

d H value.

e Not applicable

### Hospitalization and VE

There were 17,916 (33.31%) individuals hospitalized with COVID-19, and the proportion of COVID-19 in males (36.60%, 10,403/28,423) was higher than that in females (29.62%, 7513/25,366) (*P*<.001). The proportion in inpatients aged ≥90 years (44.99%, 3833/8519) was higher than that in inpatients aged 80‐89 years (31.11%, 14,083/45,270) (*P*<.001). Of these, 3535 (6.57%) were COVID-19–related hospitalization, 961 (1.79%) were COVID-19 severe or critical cases, and 4130 (7.68%) were in-hospital deaths in the study cohort. The incidence of hospitalization with COVID-19–related disease was estimated to be 2.66 % among 673 thousand people aged 80 years and older in Beijing. Most cases occurred in December and January of the following year (Figure 1).

**Figure 2.**
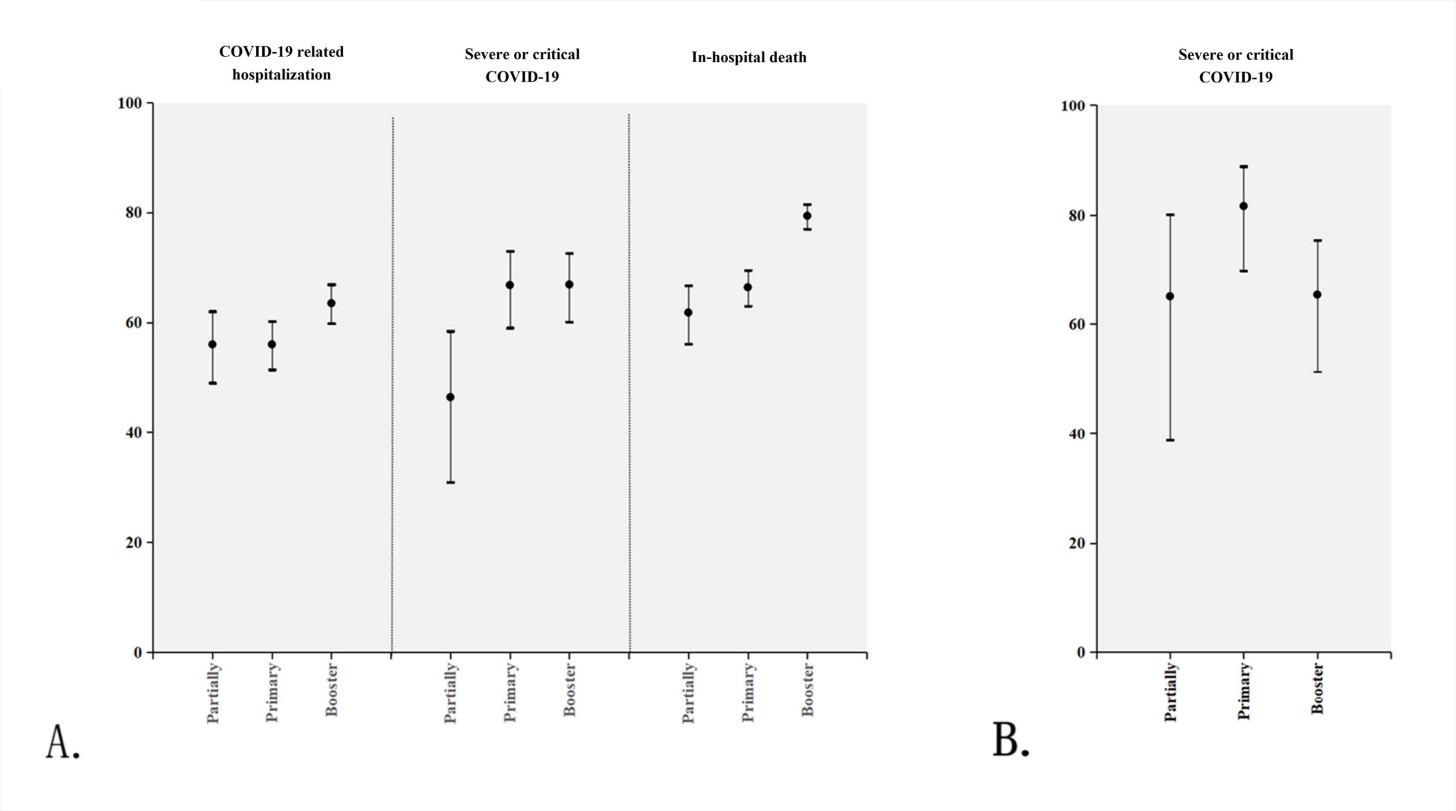
Adjusted vaccine effectiveness against severe outcomes by immunization status in adults aged ≥80 years during Omicron circulation, Beijing, China (November 2022 to February 2023) (Cohort study of hospitalized patients; vaccine effectiveness estimates via Poisson regression). (A) VE against COVID-19–related hospitalization, severe or critical disease, and in-hospital death; (B) Sensitivity analysis restricting to cases with severe or critical COVID-19 as principal diagnosis.

**Figure 3.**
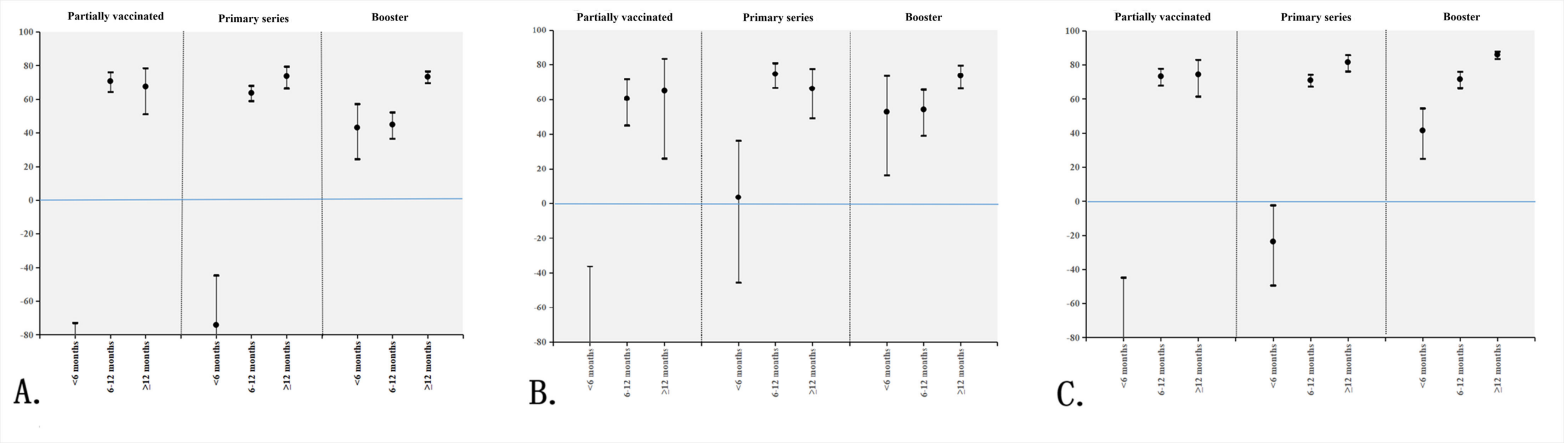
Stratified vaccine effectiveness with 95% CI by the time interval between the last vaccination dose and hospital admission for COVID-19 related outcomes among the older inpatients aged ≥80 years during the Omicron circulation (November 2022 to February 2023) in Beijing, China. (A) Hospitalization (principal diagnosis); (B) Severe or critical disease (principal or secondary diagnosis); (C) In-hospital death. Transverse lines that cross the vertical blue line at zero indicate no statistical significance.

The risk of COVID-19–related hospitalization in the unvaccinated group (9.76%, 2269 cases) was higher than that in the vaccinated group (partially vaccinated: 4.49%, 203/4524 cases; primary series: 4.49%, 505/11,246 cases; booster vaccination: 3.78%, 558/14,761 cases). The effectiveness of COVID-19 immunization in preventing COVID-19–related hospitalization was 56% (95% CI 49%‐62%), 56% (95% CI 51.4%‐60.2%), 63.5% (95% CI 59.8%‐66.9%) for partial immunization, primary series, and booster vaccination, respectively (Figure 2). Figure 3 shows the VE stratified by the time interval between the last dose and hospital admission. VE of primary series was 63.7% (95% CI 58.9%‐68%) and 73.7% (95% CI 66.5%‐79.4%) during 6‐12 months and ≥12 months, respectively. VE of booster was 43.1% (95% CI 24.4%‐57.2%) for <6 months, 44.9% (95% CI 36.5%‐52.2%) during 6‐12 months, and 73.3% (95% CI 69.6%‐76.5%) during ≥12 months.

The risk of severe or critical COVID-19 in the unvaccinated group (2.8%, 652 cases) was higher than that of the vaccinated group (0.92%‐1.48%). The effectiveness of COVID-19 vaccines against severe or critical COVID-19 was 46.4% (95% CI 30.9%‐58.4%), 66.8% (95% CI 59%‐73%) and 66.9% (95% CI 60.1%‐72.6%) for partial immunization, primary series, and booster vaccination, respectively (Figure 2). VE of primary series was 74.6% (95% CI 66.6%‐80.7%) during 6‐12 months, and 66.2% (95% CI 49%‐77.6%) ≥12 months following vaccination. VE of booster was 52.9% (95% CI 16.2%‐73.5%) for <6 months, 54.2% (95% CI 39%‐65.6%) during 6‐12 months, and 73.6% (95% CI 66.3%‐79.4%) for ≥12 months following vaccination (Figure 3).

The risk of in-hospital death with COVID-19 disease in the unvaccinated group (12.86%, 2990/23,258 cases) was higher than that of the vaccinated group (partially vaccinated: 5.11%, 231/4524 cases; primary series: 4.48%, 504/11,246 cases; booster vaccination: 2.74%, 405/14,761 cases). The effectiveness of booster vaccination against in-hospital deaths was 79.4% (95% CI 77%‐81.5%), higher than that of primary series (66.4% [95% CI 63%‐69.5%]). The effectiveness of partial COVID-19 immunization in preventing in-hospital death was 61.8% (95% CI 56.1%‐66.7%) (Figure 2). VE of primary series was 71% (95% CI 67.3%‐74.3%) during 6‐12 months, and 81.6% (95% CI 76.2%‐85.8%) ≥12 months following vaccination. VE of booster was 41.5% (95% CI 24.9%‐54.5%) for <6 months, 71.6% (95% CI 66.4%‐76%) during 6‐12 months, and 86% (95% CI 83.6%‐87.9%) for ≥12 months following vaccination (Figure 3).

While VE generally declined with time since vaccination, the point estimate for ≥12 months post-booster appeared higher than the 6‐12 months group. This anomaly might be attributable to the survivor bias, the population in this ≥12 months cohort opted for vaccination earlier (before November 2021) because they were in better health and awareness of health protection, which could lead to an overestimation of VE.

### Sensitivity Analysis

If only the older individuals with severe or critical COVID-19 in “principal diagnosis” were analyzed, the VE against hospitalization decreased to 65.1% (95% CI 38.8%‐80.1%), 81.6% (95% CI 69.8%‐88.8%), 65.4% (95% CI 51.3%‐75.4%) for partial immunization, primary series, and booster vaccination, respectively (Figure 2). The VE of the booster was comparable to the findings of the main analysis, with varying precision due to reduced case counts (n=42 vs n=138 in main analysis). A similar pattern was observed in the stratified analysis based on different vaccination intervals (Table S1 and Table S2 in Multimedia Appendix 2 for time-stratified results).

## Discussion

### Principal Findings

During an Omicron BF.7-predominant wave of COVID-19 in Beijing city, we used electronic individual-level hospitalization records matching with record-documented COVID-19 vaccination status to calculate the characteristics and vaccination status of the inpatients aged ≥80 years and estimate the real-world effectiveness of domestic COVID-19 vaccines. This study shows that the proportion of hospitalizations diagnosed with COVID-19 disease in vaccination groups was significantly lower for those who had not been vaccinated. When given according to the recommended schedule of a 2-dose primary series followed by a booster dose 6 months later, the VE against COVID-19–related hospitalization outcomes was 63.5%‐79.4%. These results represent absolute modest VE associated with vaccine-induced protection for inpatients aged ≥80 years, uninfluenced by hybrid immunity.

COVID-19–associated hospitalization rates remain higher among older adults relative to rates among younger adults, adolescents, and children [25]. The Omicron variant of SARS-CoV-2 has been reported as more transmissible, but less severe, than previous variants. In the study of all hospitalization for population aged 80 years and older, 33.31% of inpatients had COVID-19–related diagnosis, the incidence of hospitalization with COVID-19 disease in ≥80-year-old people was estimated as 2.66% in the United Kingdom [26], the proportion of older adults aged ≥80 years who were hospitalized in all COVID-19 cases with Omicron (15.2%) was highest compared with other age groups of participants (1.47%‐5.80%). Compared with other Omicron variants, the studies in New England [27] found that 3.1% of patients infected with Omicron BA.2 required admission to hospitalization for general population (The mean age is 49.5). Up to now, older adults still were listed as the highest priority groups in the COVID-19 vaccination guidance [28]. In comparison with other countries, China initiated its vaccine roll-out campaign relatively early [29]. Despite the coverage of primary series for people aged 18 years and older in Beijing having already raised to a high level before the Omicron epidemic wave and quickly carrying out the booster program, 30% of adults aged 80 years and older remained unvaccinated or partially vaccinated, only 46.7% completing the booster shots [30]. China has the highest number of older individuals globally [31]. To prevent excessive disease severity and mortality owing to vaccination hesitancy, devoting greater effort toward vaccinating the older population is warranted given the uncertainty regarding future epidemic waves with frequent emergence of virus variants.

The effectiveness of the COVID-19 vaccines has relatively well maintained over time against the Omicron variant and its sublineages for severe disease compared to pre-Omicron variants of concern [4]. Our analysis demonstrated the primary series effectiveness against hospitalization and mortality was slightly higher than that reported in Brazilian test-negative case-control studies for those aged ≥80 years who completed primary series of CoronaVac (inactivated vaccine) during the Gamma variant predominance (38.9% [21.4%‐52.5%] and 44% [20.3%‐60.6%], respectively) [32]. This difference may be due to several factors. Circulating variants and prior infection status played significant roles. Other elements, such as the time interval between vaccination and the outcome, hospitalization criteria, population distribution, and research methods can also influence the calculated VE. However, similar to the conclusions found in most studies, our estimated VE of booster against COVID-19–related hospital admissions was higher than that of the primary series. One study in China indicated that a completed vaccination with a booster reduced the risk of asymptomatic or mild Delta or Omicron variant COVID-19 progressing to pneumonia [33]. In the study of quarantined close contacts of BA.2-infected individuals in China [34], primary series VE against pneumonia or worse infection was 66% (95% CI 57%‐73%) and 91% (95% CI 91%‐96%), booster dose VE was 74% (95% CI 69%‐79%) and 93% (95% CI 87%‐97%), respectively. Among individuals 80 years and older in the McMenamin study, VE increased from 58% to 97% when boosted [15]. In a prospective cohort study across UK nations, data on 30 million people aged ≥18 years from primary care, testing, vaccination, hospitalization, and mortality were linked. They found that compared with received primary doses, the first booster of BNT162b2 (tozinameran; Pfizer-BioNTech) or mRNA-1273 (elasomeran; Moderna) could have reduced severe outcomes (hospitalization or death) due to Omicron B.1.1.529 variant (the rate decreased from 8.8 to 7.6 events per 1000 person-y) [18]. Compared with the observed VEs in aforementioned studies conducted during the stages of Alpha, Gamma, and initial Omicron predominance [15,35,36], our estimated result, which focuses on the period of Omicron BF.7 predominance and BA.5.2, was lower. This study also evaluated VE against in-hospital deaths with COVID-19 disease, but it was difficult to determine whether the deaths marked among inpatients with the U07.1 *ICD-10* code were actually caused by COVID-19 disease, so the direct protective effect could not be calculated due to many confounding factors (such as the reason for hospitalization, treatment measures, etc). Moreover, in line with previous research, a decline in VE was observed as age increased. During the Omicron BA.2 subvariant wave in Hong Kong, VE against severe or fatal disease was 96.3% (94.9%‐97.3%) for 2 doses of BNT162b2 and 91.7% (88.7%‐94%) for 2 doses of CoronaVac in those aged 20‐59 years, reducing to 86.9% (80.5%‐91.3%) and 58.2% (45.1%‐68.2%) among those aged 80 years or older [15]. A parallel pattern emerged in a Shanghai Omicron BA.2 outbreak, where 3 doses of inactivated vaccines showed 96.0% (91%‐98.3%) effectiveness against severe or critical COVID-19 in the 60‐69 age cohort, decreasing to 66.8% (−22.5% to 91%) among people aged ≥90 years [16]. Older people have a less complete immune response to antigens and a reduced ability to develop robust immunity after infections or vaccination because of the age-related decline in the functioning of the immune system [37].

For the primary and booster immunizations of the COVID-19–inactivated vaccine, the GMT against the wild-type strain declined gradually during the 12 months after the primary immunization and was lower against the Delta and Omicron variant spike proteins [38]. It is necessary to estimate the protective effect of different intervals after the latest vaccination and how long vaccinated individuals should be evaluated before future epidemic trends. As of November in 2022, the COVID-19 mass vaccination has completed and most people have gotten the latest dose for 12 months and longer. There were too few COVID-19 cases with 6 months between the last dose and exposure to estimate reliable VEs stratified by time; results are shown in Supplementary Table S2 in Multimedia Appendix 2. VE of vaccination in more than 12 months and during 6 months and 12 months after the last dose has remained 52%‐86%. The highest VE observed in the ≥12-month post-vaccination group contradicts established immune waning patterns. This paradox may reflect methodological biases, one is healthy survivor bias, the population in this ≥12-month cohort opted for vaccination earlier on because they were in better health and awareness of health protection, or the healthiest individuals survived long enough to be in the ≥12 months group, all these could lead to an overestimation of VE. Additionally, temporal confounding may have occurred, the ≥12 months group may have been infected later in the wave when hospital triage and treatment protocols improved, or the virus variant and the population’s immunity status may have changed over the study period. In addition, as more than 4 doses of vaccine have been administered worldwide, it is necessary to further track and monitor the vaccination situation and VE in China.

### Strengths and Limitations

One of the strengths of this study is the effective linkage between hospital admission data and vaccination records, which enables a low-cost and large-scale real-world evaluation of vaccine effects in the population. Specifically, this approach provides valuable insights into the effectiveness of prototype inactivated COVID-19 vaccines in protecting the eldest adults. The other one is our results reveal that, among inpatients aged 80 years and above who have not had a previous SARS-CoV-2 infection, there is a moderately significant level of protection. Importantly, this protection remains independent of hybrid immunity. In the context of multiple global epidemics, especially those involving Omicron variant strains, most people have experienced one or more infections. When evaluating the protective effect of vaccination against Omicron, it is difficult to exclude the confounding effects of previous infections with SARS-CoV-2 variants. However, this study still offers unique evidence for the basic protective effect of inactivated vaccines.

Our study had several limitations. First, the foremost concern is unmeasured confounding from fundamental health status differences between vaccinated and unvaccinated groups. The unvaccinated group was significantly older (86 vs 83 y) and had more females (49.60% vs 39.87%; *P*<.001) than the booster group, suggesting poorer baseline health or frailty limiting vaccine access. Critically linked is our inability to stratify by underlying diseases, a key unmeasured confounder. While we adjusted for available confounders (age and sex), unmeasured factors may persist, and the VE estimates may be influenced by unmeasured health status differences between the groups, as the unvaccinated group’s inherently poorer health may lead to worse outcomes independent of vaccination status. Future studies should use targeted designs that prospectively enroll subgroups stratified by specific comorbidities, implement propensity score matching to further mitigate confounding. Second, use of all-cause in-hospital mortality among patients diagnosed with COVID-19 cannot represent or estimate the specific COVID-19 directly caused by COVID-19. This misclassification could introduce bias into the estimates of VE against such in-hospital deaths. Third, there might have been potential selection bias in our cohort design without nonhospitalized individuals. Considering the hospitalized population may have worse physical conditions than those of the nonhospitalized population, the evaluation based on hospitalized cases may underestimate the value of VE. During the establishment of the cohort, eliminating individuals who were vaccinated during the study period would introduce a selection bias to the results, which cannot represent the true situation of the population. The composition of the inpatients in this study was close to the age distribution of permanent residents in Beijing (83.09% were 80‐89 y old, 16.91% were ≥90 y old), but the difference in gender composition (56.24% were female and 43.76% were male), although estimated VE was adjusted for age and gender would decrease this bias, our results still can only be limited to representing the hospitalized population, may not be generalizable to nonhospitalized or younger populations. Another point to consider is that, compared with experimental studies, the observational study cannot absolutely control external conditions; therefore, there could be unmeasured confounding variables.

### Conclusions

In conclusion, booster vaccination was associated with significantly reduced risk of COVID-19–related severe or critical illness or in-hospital deaths in older inpatients aged ≥80 years during an Omicron-dominant period. Offering a booster dose and regularly monitoring the coverage and VE of COVID-19 vaccines remains the mainstay for mitigating impacts of upcoming epidemics. While our results demonstrate a significant association between booster vaccination and reduced severe outcomes, these estimates may reflect both VE and the better baseline health status of the vaccinated older individuals.

## Supplementary material

10.2196/82915 Multimedia Appendix 1 Flow chart of the study cohort.

10.2196/82915 Multimedia Appendix 2 Vaccine effectiveness of COVID-19 vaccines considering different immunization status, intervals since the last dose against COVID-19–related hospitalization during the Omicron wave in Beijing, along with characteristics of hospitalized patients in the cohort.

## Abbreviations

BHUIS: Beijing Hospital Utilization Information System
BMHCIC: Beijing Municipal Health Commission’s Information Center
*ICD-10*: *International Statistical Classification of Diseases and Related Health Problems 10th Revision*
IIS: immunization information system
VE: vaccine effectiveness
