# Supplementary figures and images for: Effectiveness of Inactivated COVID-19 Vaccination Against COVID-19–Related Hospitalization and Severe Outcomes in Adults ≥80 Years During Omicron Circulation in Beijing, China: Retrospective Cohort Study
Source: JMIR Public Health Surveill. 2026 Mar 11;12:e82915. doi: 10.2196/82915 (PMC12978535; doi:10.2196/82915)

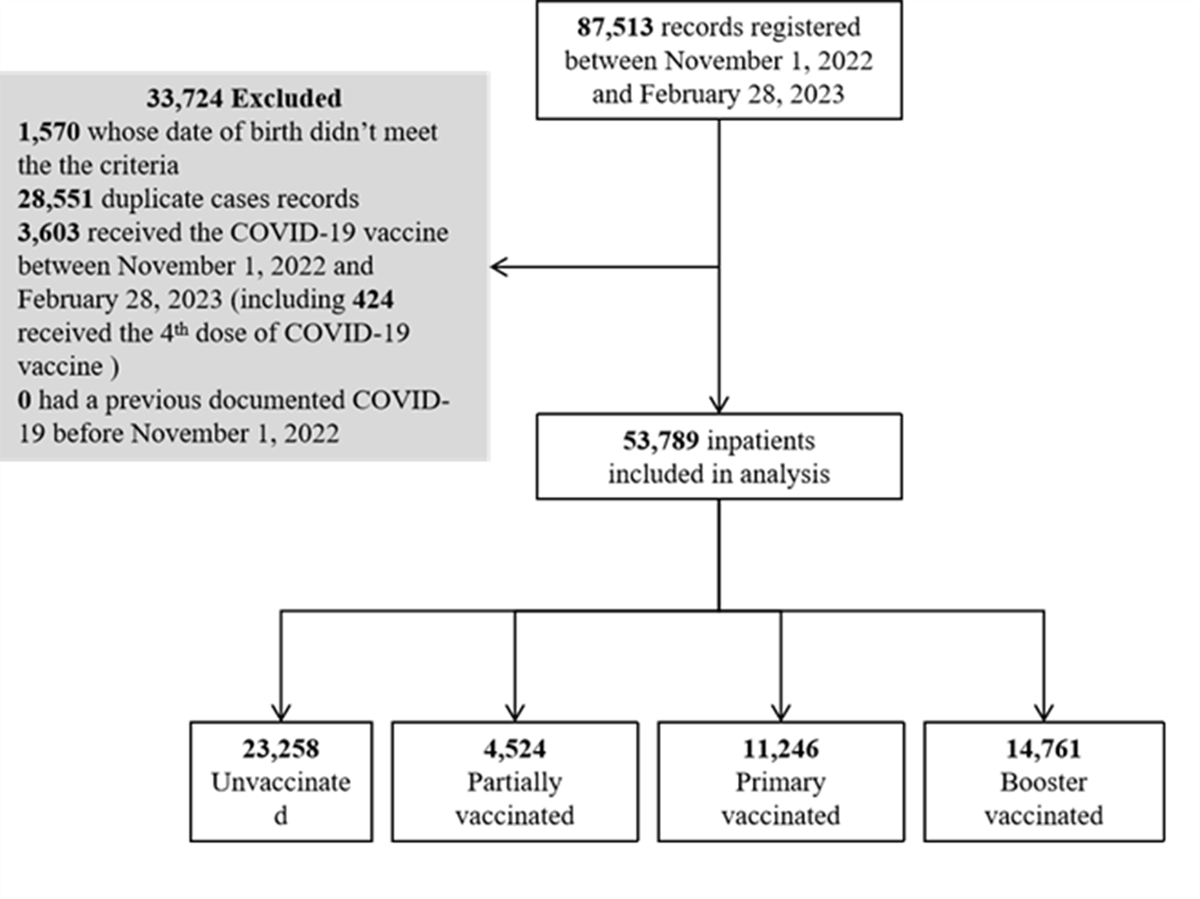

Supplement: Multimedia Appendix 1 [file publichealth-v12-e82915-s001.png]
