# Supplementary material for: Effectiveness of Inactivated COVID-19 Vaccination Against COVID-19–Related Hospitalization and Severe Outcomes in Adults ≥80 Years During Omicron Circulation in Beijing, China: Retrospective Cohort Study
Source: JMIR Public Health Surveill. 2026 Mar 11;12:e82915. doi: 10.2196/82915 (PMC12978535; doi:10.2196/82915)
Supplement: Multimedia Appendix 2 [file publichealth-v12-e82915-s002.docx]

**Table S1.** Vaccine effectiveness of different immunization histories of COVID-19 vaccines on COVID-19 disease in hospitalized population during the Omicron wave in Beijing, China;

| Outcome | Vaccination status | N | Risk  （%） | Adjusted Risk Ratio(95%CI) | VE（95%CI） |
| --- | --- | --- | --- | --- | --- |
| ***Labeled in principal diagnosis or secondary diagnosis*** | | | | | |
| COVID-19  related hospitalization | unvaccinated | 11977 | 51.50 | reference | reference |
|  | vaccinated | 5939 | 19.45 | 0.23 | 76.7(75.7,77.6) |
|  | Partially vaccinated | 1004 | 22.19 | 0.27 | 72.7(70.6,74.7) |
|  | Primary Series | 2398 | 21.32 | 0.26 | 74.0(72.6,75.3) |
|  | Booster | 2537 | 17.19 | 0.20 | 80.0(78.9,81.0) |
| Severe and critical COVID-19 | unvaccinated | 652 | 2.80 | reference | reference |
|  | vaccinated | 309 | 1.01 | 0.36 | 63.8(58.4,68.5) |
|  | Partially vaccinated | 67 | 1.48 | 0.54 | 46.4(30.9,58.4) |
|  | Primary Series | 104 | 0.92 | 0.33 | 66.8(59.0,73.0) |
|  | Booster | 138 | 0.93 | 0.33 | 66.9(60.1,72.6) |
| ***Labeled in principal diagnosis*** | | | | | |
| COVID-19  related hospitalization | unvaccinated | 2269 | 9.76 | reference | reference |
|  | vaccinated | 1266 | 4.15 | 0.41 | 59.5(56.5,62.4) |
|  | Partially vaccinated | 203 | 4.49 | 0.44 | 56.0(49.0,62.0) |
|  | Primary Series | 505 | 4.49 | 0.44 | 56.0(51.4,60.2) |
|  | Booster | 558 | 3.78 | 0.37 | 63.5(59.8,66.9) |
| Severe and critical COVID-19 | unvaccinated | 198 | 0.85 | reference | reference |
|  | vaccinated | 72 | 0.24 | 0.29 | 71.4(62.4,78.3) |
|  | Partially vaccinated | 13 | 0.29 | 0.35 | 65.1(38.8,80.1) |
|  | Primary Series | 17 | 0.15 | 0.18 | 81.6(69.8,88.8) |
|  | Booster | 42 | 0.28 | 0.35 | 65.4(51.3,75.4) |
| ***In-hospital death with COVID-19 disease*** | | | | | |
|  | unvaccinated | 2990 | 12.86 | reference | reference |
|  | vaccinated | 1140 | 3.73 | 0.28 | 71.8(69.7,73.8) |
|  | Partially vaccinated | 231 | 5.11 | 0.38 | 61.8(56.1,66.7) |
|  | Primary Series | 504 | 4.48 | 0.34 | 66.4(63.0,69.5) |
|  | Booster | 405 | 2.74 | 0.21 | 79.4(77.0,81.5) |

**Table S2.** Vaccine effectiveness of COVID-19 vaccines by intervals since the last dose against COVID-19 disease in hospitalized population during the Omicron wave in Beijing, China

| Vaccination status | **Intervals since the last dose** | **Labeled in principal diagnosis and secondary diagnosis** | |  | **Labeled in principal diagnosis** | |  | **In-hospital death with COVID-19 disease** |
| --- | --- | --- | --- | --- | --- | --- | --- | --- |
|  |  | COVID-19 related hospitalization | Severe and critical COVID-19 |  | COVID-19 related hospitalization | Severe and critical COVID-19 |  |  |
| **Vaccinated** | <6 months | -35.2(-48.0,-23.5) | 4.6(-25.3,27.4) |  | -29.9(-48.8,-13.4) | **40.2(-9.9,67.5)** |  | -5.2(-20.0,7.8) |
|  | 6-12 months | 78.0(76.9,79.1) | 65.8(59.0,71.5) |  | 60.2(56.4,63.7) | 72.3(60.3,80.7) |  | 71.7(68.9,74.2) |
|  | ≥12 months | 84.6(83.6,85.4) | 71.6(64.9,77.0) |  | 72.9(69.7,75.8) | 75.9(63.4,84.1) |  | 84.6(82.4,86.4) |
| **Partially vaccinated** | <6 months | -852.9(-1257.5,-568.9) | -111.2(-227.3,-36.3) |  | -123.8(-189.7,-73.0) | -53.4(-274.9,37.3) |  | -86.0(-138.8,-44.8) |
|  | 6-12 months | 82.6(80.8,84.2) | 60.5(45.1,71.6) |  | 70.7(64.3,76.0) | 75.5(47.8,88.5) |  | 73.3(67.9,77.8) |
|  | ≥12 months | 78.4(73.8,82.2) | 64.9(25.7,83.4) |  | 67.5(51.1,78.4) | 83.0(-21.9,97.6) |  | 74.4(61.4,83.0) |
| **Primary Series** | <6 months | -132.8(-170.1,-100.6) | 3.5(-45.8,36.2) |  | -74.2(-109.8,-44.7) | 86.8(5.9,98.2) |  | -23.7(-49.4,-2.4) |
|  | 6-12 months | 79.7(78.4,81.0) | 74.6(66.6,80.7) |  | 63.7(58.9,68.0) | 84.7(71.2,91.9) |  | 71.0(67.3,74.3) |
|  | ≥12 months | 81.7(79.6,83.7) | 66.2(49.0,77.6) |  | 73.7(66.5,79.4) | 71.3(35.1,87.3) |  | 81.6(76.2,85.8) |
| **Booster** | <6 months | 53.3(46.1,59.5) | 52.9(16.2,73.5) |  | 43.1(24.4,57.2) | 33.8(-61.3,72.9) |  | 41.5(24.9,54.5) |
|  | 6-12 months | 70.0(67.5,72.2) | 54.2(39.0,65.6) |  | 44.9(36.5,52.2) | 46.2(12.3,66.9) |  | 71.6(66.4,76.0) |
|  | ≥12 months | 85.6(84.6,86.5) | 73.6(66.3,79.4) |  | 73.3(69.6,76.5) | 76.8(62.5,85.6) |  | 86.0(83.6,87.9) |

*Unvaccinated as the reference.

**Table S3.** Basic characteristics of hospitalized with COVID-19 related disease in study cohort

| **Characteristics** | Total, no.(%) | hospitalized with COVID-19 related disease  no.(proportion,%) | Chi-  Square/Z value | P  value |
| --- | --- | --- | --- | --- |
| no. of persons | 53,789 | 17,916(33.31) |  |  |
| Sex |  |  |  |  |
| Male | 28423 (52.84) | 10,403(36.60) | 294.17 | <0.001 |
| Female | 25366(47.16) | 7,513(29.62) |  |  |
| Median age, years (IQR) | 84 (82-86) | 86 (83-89) | -31.88 | <0.001 |
| Age group, years |  |  | 622.23 | <0.001 |
| 80~89 | 45270(84.16) | 14,083(31.11) |  |  |
| 90+ | 8519(15.84) | 3,833(44.99) |  |  |
| COVID-19 vaccine status |  |  |  |  |
| Unvaccinated | 23258(43.24) | 11,977(51.50) | 6169.34 | <0.001 |
| Partially vaccinated | 4524(8.41) | 1,004(22.19) |  |  |
| Primary vaccination | 11246(20.91) | 2,398(21.32) |  |  |
| Booster vaccination | 14761(27.44) | 2,537(17.19) |  |  |
